# Predicting avoidant coping in individuals recently diagnosed with serious illness: a cross-sectional study

**DOI:** 10.3389/fpsyg.2026.1773360

**Published:** 2026-03-09

**Authors:** Isabella Rasthøj Holst, Sussi Friis Buhl, Trine Thilsing, Maria Munch Storsveen, Sonja Wehberg, Tina Birgitte Wisbech Carstensen, Dorte Ejg Jarbøl

**Affiliations:** 1 The Research Unit of General Practice, Department of Public Health, University of Southern Denmark, Odense, Denmark; 2 Department of Functional Disorders, Aarhus University Hospital, Aarhus, Denmark; 3 Department of Clinical Medicine, Aarhus University, Aarhus, Denmark; 4 Department of Anthropology, School of Culture and Society, Aarhus University, Aarhus, Denmark

**Keywords:** avoidance, coping, prediction model, recently diagnosed, serious illness, social vulnerability

## Abstract

**Introduction:**

Avoidant coping has been linked to poorer health outcomes. This cross-sectional study aimed to examine whether high avoidant coping can be reliably predicted from health parameters and socioeconomics among adults recently diagnosed with a serious illness.

**Methods:**

A nationwide survey linked to national registers. Inclusion criteria were: (i) age ≥50 years, and (ii) diagnosed with cancer, neurological disease, and/or heart disease within the year preceding the survey. Coping was assessed using the Brief Approach/Avoidance Coping Questionnaire with high avoidance defined as > mean avoidance score +1 SD. Predictive models were developed using data from all survey respondents aged ≥50 years and subsequently tested in the recently diagnosed subsample. Area Under the Curve (AUC) and 95% Confidence Intervals (CI) were reported.

**Results:**

The recently diagnosed sample comprised 746 individuals, of whom 13.4% exhibited high avoidant coping. High avoidance was associated with female sex, lower educational level, shorter self-reported life-expectancy, and poorer perceived social support. However, the predictive models demonstrated poor discriminative capacity (AUC 0.62; CI: 0.57–0.68) for the recently diagnosed sample. Among adults aged 50 + years recently diagnosed with serious illness, high avoidance could not be reliably predicted from health parameters and socioeconomics.

## 1 Introduction

The incidence of serious illnesses such as cancer, heart disease, and neurological disease increases with increasing age (Townsend et al., 2022; Deuschl et al., 2020; Dyba et al., 2021), and the adaptational process of living with a serious illness can be challenging for the individual. Being diagnosed with a serious illness may interfere with daily living and create a need to deal with a range of stressors such as concern, pain, medical treatment, impaired functional status, decreased quality of life, and altered social relations (Stanton et al., 2007; Benkel et al., 2019; Smith et al., 2016; Andersen, 2024).

Individuals may react and adapt differently in this transitional phase following a recent diagnosis of a serious illness, which can be explained within the framework of the widely accepted psychological Common-Sense Model of Self-Regulation (CSM). The CSM provides a conceptual framework for examining the perceptual, behavioural, and cognitive processes involved in the individuals’ self-management of a health threat such as serious illness (Leventhal et al., 2016). According to the CSM, individuals’ responses are based on prototypes of illness that include expectations regarding illness identity, timeline, causes, consequences, and personal control. These components form an individual’s cognitive representation of the illness, that is, our common sense beliefs that serve as a mental framework for understanding the condition. This framework directly influences how individuals cope with their illness, e.g., how they navigate in the health care system (e.g., seeking treatment, advice, support), manage their illness and react to symptoms, and/or change their behaviour to adapt to their altered life conditions (Leventhal et al., 2016; Diefenbach and Leventhal, 1996). Differences in coping behaviours may be some of the reasons why there is considerable variability in adjustment to illness (Stanton et al., 2007) and why individuals report different levels of pain, disability, and fatigue despite having similar clinical parameters (Stone et al., 1997; Leboeuf-Yde et al., 2011; Mallinson et al., 2006). In addition to previous experiences with illness, coping responses are also formed by personal characteristics such as personality, personal resources (e.g., social support, optimism, mastery, and self-esteem), and experiences of negative affect (e.g., anxiety and depression) (Taylor and Stanton, 2007; Carver and Connor-Smith, 2010; Lazarus, 1984).

Literature shows that some coping behaviours are more efficient than others. In the acute phase after the onset of the stressor, avoidance, such as denial and neglect may be adaptive (e.g., resting behaviour, taking pain killers) (Suls and Fletcher, 1985; Nelson et al., 2022). However, avoidance has repeatedly been shown to be associated with poorer long-term health outcomes (Suls and Fletcher, 1985; Nelson et al., 2022; Raasthøj et al., 2024; Penley et al., 2002; Livneh, 2019) in different patient groups such as patients with cancer (O'Brien and Moorey, 2010), Parkinson’s disease (Evans and Norman, 2009), multiple sclerosis (Culicetto et al., 2024), and heart disease (Klein et al., 2007). In contrast to avoidant coping, approach coping, such as problem-solving and seeking social support have been considered more adaptive in the context of illness (Suls and Fletcher, 1985; Raasthøj et al., 2024). Accordingly, identifying individuals at risk of avoidant coping, particularly those who have recently been diagnosed with a serious illness, is important.

Despite the many studies examining the efficacy of coping in relation to various outcomes, only few studies have described the characteristics of individuals with an avoidant coping profile (Raasthøj et al., 2024; Sætre et al., 2023; Carstensen et al., 2012). Based on these studies, health parameters and socioeconomics may be of importance. Women and individuals with poorer health status and lower educational level tend to use avoidant coping more frequently (Raasthøj et al., 2024; Sætre et al., 2023) whereas living with a partner and being employed are associated with a reduced use of avoidance coping (Kildal et al., 2005). Notably, women also employ approach coping strategies more often than men, whereas findings related to age are ambiguous (Sætre et al., 2023). Although evidence suggest a link between coping and socioeconomic parameters and health status, it remains unclear if such characteristics can be used to identify individuals with avoidant coping.

Gaining deeper insight into the characteristics of individuals aged 50 years and above who have recently been diagnosed with a serious illness and exhibit high avoidant coping may help healthcare professionals to identify those who require additional support to manage the challenges associated with newly diagnosed serious illness. Based on a sample from the general Danish population, the purpose of this study was to (i) describe characteristics of 50 + years old individuals who have recently been diagnosed with a serious illness, (ii) identify and characterise individuals within this group who report high avoidant coping, and (iii) examine if high avoidance can be reliably predicted from health parameters and/or socioeconomics in (a) the total sample of 50 + year old individuals and (b) among 50 + year old individuals who have recently been diagnosed with serious illness.

## 2 Materials and methods

### 2.1 Study design and populations

A cross-sectional study was carried out among individuals who participated in the Danish Symptom Cohort (DaSC) II study, which is a large population-based survey from 2022 with the overall aim of examining symptoms and healthcare seeking in the Danish general population. The methodological frameworks of the DaSC II survey have been described in detail elsewhere (Sætre et al., 2024; Elnegaard et al., 2015; Rasmussen et al., 2014). In brief, the study sample for the DaSC II survey comprised 100,000 adults aged 20 years or older who were randomly selected from the general population using the Danish Civil Registration System (Mainz et al., 2019). Data collection took place from May to July 2022. All invitees received an invitation in their personal digital mailbox (e-Boks), which is used for secure communication between public authorities, citizens, and businesses in Denmark (e-Boks, n.d.).

The conceptual framework and the questionnaire development are described in detail elsewhere (Sætre et al., 2024), but overall, the development of the questionnaire for the DaSC II survey followed the COnsensus-based Standards for the selection of health Measurement INstruments (COSMIN) guidelines (Mokkink et al., 2010). The survey was designed with a forced answer feature and leap structure to omit missing responses and unnecessary questions, respectively.

For the present study we operationalised two populations from the DaSC II study:

1. Total study sample: Respondents who were 50 + years at the time of data collection.
2. Recently diagnosed sample: Respondents who were 50 + years at the time of data collection and had a recent diagnosis of cancer, cardiac, and/or neurological disease (detailed in section 2.3)

Respondents from the DaSC II study were excluded if they were younger than 50 years at the time of data collection and if they had missing or incomplete data on coping.

### 2.2 Ethics approval and consent to participate

Participants invited to the DaSC II survey received a detailed invitation letter outlining the purpose of the study, the content and nature of the questionnaires, and the legal basis for data processing. It was clearly stated that by completing the survey, participants provided informed consent for the use of their data for research purposes in accordance with the Danish Data Protection Act, §10 (https://www.retsinformation.dk/eli/lta/2018/502). Participation was entirely voluntary, and invitees were informed of their right to decline participation without any consequences. Respondents were also advised that no clinical follow-up would be provided. Additionally, participants were given the opportunity to contact the research team via phone or email for further information or clarification. The project was approved by the Research Ethics Committee at University of Southern Denmark (Case no. 21/29156) and has been registered by the Danish Data Protection Agency (J. no. 2011-41-6651) through the Research and Innovation Organisation (RIO), University of Southern Denmark (Project number 10.104). Reporting of the study followed the STROBE guideline (Cuschieri, 2019).

### 2.3 Recently diagnosed sample

The target group of interest for the present study comprised individuals 50 + years who were recently diagnosed with cancer, neurological disease, and/or heart disease. The term *recently* referred to the 12-month period preceding the questionnaire assessment. Cancer, neurological disease, and heart disease were chosen based on the following criteria: (i) serious and/or progressive conditions, (ii) that require contact with a hospital, and/or (iii) affect daily living in terms of treatment, medication, symptoms, etc. Data from the Danish National Patient Registry that contains individual-level nationwide data on diagnoses according to the International Classification of Diseases, 10th edition (ICD 10) was used (Mainz et al., 2019; Schmidt et al., 2015). All hospital contacts are registered in the Danish National Patient Registry with a primary diagnosis as well as secondary diagnoses when relevant (Schmidt et al., 2015). Diagnoses from general practice and private practice specialists are not included in this register (Schmidt et al., 2015). To ensure the identification of individuals with newly onset disease and to avoid including cases that represent exacerbations of pre-existing conditions, individuals with a registered diagnosis of cancer, neurological disease, or heart disease within the 10 years preceding the 12-month registration period were excluded.

For cancer, all types (DC00-DC98) were selected except ‘DC44 Other malignant neoplasms of skin’, which is typically a localised, slowly progressing, and highly curable cancer (Gordon, 2013). Diagnoses of cardiovascular and neurological disease were selected in a two-step process. First, two members of the author group (IRH and SFB) reviewed the Danish version of the ICD-10 chapter VI Diseases of the nervous system and chapter IX Diseases of the circulatory system (Sundhedsdatastyrelsen, 2025a; Sundhedsdatastyrrelsen, 2025b). Relevant diagnoses were noted on a list, and if the two members were in doubt or disagreed, the list was reviewed by another member of the project group (DEJ), and the disagreements were resolved by a joint discussion. The final list of ICD 10 diagnoses used for defining the subgroup of individuals recently diagnosed with serious illness can be found in Supplementary Table S1.

### 2.4 Coping

Coping was measured by the Brief Approach/Avoidance Coping Questionnaire (BACQ) that was originally developed by Finset et al. (2002) to measure a general concept of approach versus avoidance-oriented coping, not restricted to health issues and disease. This questionnaire was chosen because it provides a multidimensional assessment and captures general coping in the approach-avoidance dichotomy with only 12-items and aligns conceptually with our aim to examine general coping tendencies in individuals newly diagnosed with serious illness. The BACQ has been translated, culturally adapted, and validated to be used among adults in the Danish general population (Sætre et al., 2023). Evidence support the construct validity, internal consistency, and content validity as well as the ability to differentiate coping in subgroups with poorer health profiles (Sætre et al., 2023). The BACQ consist of three subscales of approach (items 1–6), diversion (items 7–9), and resignation (items 10–12). Respondents were asked how they would usually act in response to problems and stressful situations. The questions were written in first person, i.e., “I say so if I am angry or sad” (approach item), “I try to forget my problems” (diversion item), and “I withdraw from other people when things get difficult” (resignation item). The questions were answered on a 5-point Likert scale (5 = agree completely, 4 = tend to agree, 3 = yes and no, 2 = tend to disagree, and 1 = disagree completely). The full BACQ questionnaire with answer categories is available in Supplementary Table S2. The approach sum score ranged from 6 to 30, while the score ranged from 3 to 15 for diversion and resignation (Sætre et al., 2023). As described by Finset et al. (2002) coping assessed by the BACQ is understood within a framework contrasting approaching with avoiding stressors, reflecting a general quality in the individual’s adaptation to stress. On this theoretical basis, and consistent with the original validation studies, we operationalised avoidant coping as the total sum of diversion and resignation subscales. The total avoidance score therefore ranged from 6 to 30, and a high avoidance was operationalised as coping scores above the cut point of the mean plus one Standard Deviation (SD) estimated in the total study sample. Under the assumption of normality, 15.9% of the population would be expected to be above this threshold.

### 2.5 Covariates

Covariates were selected based on current evidence showing that coping resources can be influenced by socioeconomics and health parameters (Taylor and Stanton, 2007; Lazarus, 1984; Raasthøj et al., 2024; Sætre et al., 2023). Covariates describing socioeconomic status included register data on living situation, ethnicity, and educational level. The register data were pertained to the year before the survey (i.e., 2021). Sex (female, male), age (50–59, 60–69, 70–79, 80 + years), living situation (living alone, cohabitating), and ethnicity (Danish, immigrants/descendants) were obtained from the Danish Civil Registration System (Schmidt et al., 2014), while data on highest educational level was obtained from the Danish Education Register (Jensen and Rasmussen, 2011) and categorised into low, medium, and high level of education corresponding to <10 years, 10–15 years and >15 years of education, respectively. If register data was missing, individuals were assigned to the most prevalent group (prevalence based on the 100,000). The Nordic Multimorbidity Index (Kristensen et al., 2022) was calculated based on register data from the Danish National Patient Registry (Schmidt et al., 2015) and the Danish National Prescription Registry (Pottegård et al., 2017). Multimorbidity index was reported in four categories: (i) no multimorbidity (score≤0), and tertiles of (ii) low, (iii) medium, and (iv) high levels of multimorbidity. A full overview of variables based on register data is available in Supplementary Table S2.

Health parameters also included questionnaire data from the DaSC II survey regarding self-rated health, functional capacity, concern for own health, and life expectancy. In addition, we incorporated questionnaire data to ascertain whether respondents had access to a personal network that could accompany them to medical appointments. This aspect, referred to as *social support accompanying medical appointments*, was assessed through a single item within the fourth domain of the Health Literacy Questionnaire (Osborne et al., 2013). It is important to note that this item serves solely as a source of contextual information and is not considered a component of health literacy. The specific questions, original response categories, operationalisation, and naming in the present study are listed in Supplementary Table S2.

### 2.6 Statistical analyses

Characteristics of study populations were presented as number of observations (n) and percentage of group (%) or mean ± SD for categorical and continuous variables, respectively. A multivariable logistic regression model was used to model associations between descriptive variables and being recently diagnosed (yes/no). As a basic (first) analysis with respect to avoidant coping, we estimated odds ratios (OR) in both univariable and multivariable logistic regression models, separately for the total study sample and for the recently diagnosed sample. To address the question whether high avoidance in general can be reliably predicted from health parameters and/or socioeconomics, we then developed three different discrimination (prediction) models based on the total study sample. Baseline Model A - adjusted for age and sex; Model B - adjusted for age, sex, and socioeconomics, i.e., living situation, ethnicity, educational level, and multimorbidity index; Model C - adjusted for age, sex, self-rated health, functional capacity, concern for own health, life-expectancy, and social support during medical appointments.

These prediction models were both applied to the full development sample (total study sample) and the subset of recently diagnosed individuals. We did not employ an independent dataset for testing but illustrate the model’s predictive ability in a subset of the development sample. The ability to discriminate between individuals with and without high avoidance was illustrated in Receiver Operating Characteristic (ROC) curves, and the Area under the ROC curve (AUC) was calculated. The calculated AUC ranged from 0.5 to 1 where 0.5 corresponds to no discriminative ability (random) and 1 corresponds to perfect discrimination (Hosmer et al., 2013). Furthermore, predicted probabilities with and without high avoidance were illustrated in box plots for each sample. Measures of performance included model chi^2^, *p*-values for likelihood ratio tests comparing model A to model B and model C, the scaled Brier score (percentage, with 100% as optimal), and Integrated Discrimination Improvement (IDI) (percentage) (Steyerberg et al., 2010). Specificity, sensitivity, positive and negative predictive values were calculated to illustrate the ability of the models to correctly classify individuals into groups with and without high avoidant coping. Statistical significance was set at *p* < 0.05. Statistical analyses were performed in Stata (StataCorp. 2023. Stata Statistical Software: Release 18. College Station, TX: StataCorp LLC).

## 3 Results

### 3.1 Characteristics of the study populations

**Figure 1:**
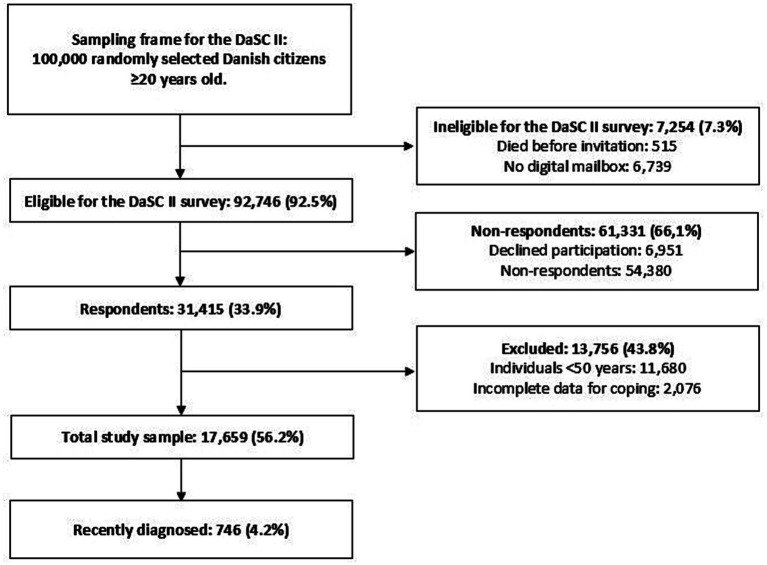
Flow chart of study populations. Flow chart includes the number of individuals and percentage (%) of the preceding group. The Danish Symptom Cohort (DaSC) II study.

Figure 1 presents the flowchart of the study populations. The sampling frame for the DaSC II survey consisted of 100,000 randomly selected individuals aged 20 years and above. Of the survey respondents (*n* = 31,415), 56.2% met the eligibility criteria for inclusion in the overall study sample. Within this group, 4.2% had received a diagnosis of cancer, cardiovascular disease, and/or neurological disease in the year preceding the data collection and were classified as the recently diagnosed sample.

Being male was associated with significantly higher odds of belonging to the recently diagnosed sample (adjusted OR 1.88, CI 1.61–2.19) (Table 1). Furthermore, individuals who were recently diagnosed with serious illness were more often of older age, had a high degree of multimorbidity, poor self-rated health, reduced functional capacity, concern about personal health, and shorter perceived life expectancy compared with individuals without a recent diagnosis of serious illness (Table 1). Socioeconomic characteristics did not differ between groups (Table 1). Crude estimates are available in Supplementary Table S3.

**Table 1:** Descriptive characteristics of the study samples and multivariable logistic regression model.

Variables	Total study sample	Recently diagnosed sample		Associations between descriptive characteristics and recent diagnosis
	*n* (column %)	*n* (column %)	row %	Adj. OR (95% CI)
Total	17,659 (100)	746 (100)	4.2	
Sex				
Female	9,550 (54.1)	293 (39.3)	3.1	Ref.
Male	8,109 (45.9)	453 (60.7)	5.6	1.88 (1.61–2.19)
Age (years)				
50–59	5,930 (33.6)	119 (16.0)	2.0	Ref.
60–69	6,032 (34.2)	237 (31.8)	3.9	1.92 (1.54–2.41)
70–79	4,562 (25.8)	291 (39.0)	6.4	3.00 (2.40–3.73)
80+	1,135 (6.4)	99 (13.3)	8.7	3.77 (2.84–4.99)
Living situation				
Cohabitating	12,870 (72.9)	537 (72.0)	4.2	Ref.
Living alone	4,789 (27.1)	209 (28.0)	4.4	1.04 (0.88–1.23)
Ethnicity				
Danish	16,785 (95.1)	715 (95.8)	4.3	Ref.
Immigrants/descendants	874 (4.9)	31 (4.2)	3.5	0.92 (0.64–1.34)
Educational level				
Low	1,578 (8.9)	83 (11.1)	5.3	Ref.
Medium	9,221 (52.2)	399 (53.5)	4.3	1.15 (0.89–1.47)
High	6,860 (38.8)	264 (35.4)	3.8	1.10 (0.85–1.43)
Multimorbidity index				
No multimorbidity	11,359 (64.3)	352 (47.2)	3.1	Ref.
Low	2,927 (16.6)	135 (18.1)	4.6	1.54 (1.25–1.89)
Medium	1,569 (8.9)	98 (13.1)	6.2	2.09 (1.65–2.64)
High	1804 (10.2)	161 (21.6)	8.9	2.86 (2.34–3.48)
Self-rated health				
Excellent	6,113 (34.6)	156 (20.9)	2.6	0.70 (0.57–0.85)
Good	8,209 (46.5)	341 (45.7)	4.2	Ref.
Poor	3,337 (18.9)	249 (33.4)	7.5	1.64 (1.37–1.96)
Functional capacity				
Good functional capacity	12,477 (70.7)	445 (59.7)	3.6	Ref.
Fair functional capacity	3,848 (21.8)	214 (28.7)	5.6	1.42 (1.19–1.69)
Poor functional capacity	1,334 (7.6)	87 (11.7)	6.5	1.43 (1.11–1.85)
Concern for own health				
Not/slightly concerned	13,294 (75.3)	442 (59.2)	3.3	Ref.
Moderately concerned	2,752 (15.6)	163 (21.8)	5.9	1.68 (1.39–2.04)
Highly concerned	1,613 (9.1)	141 (18.9)	8.7	2.32 (1.88–2.87)
Life-expectancy				
Longer life-expectancy	5,302 (30.0)	177 (23.7)	3.3	0.74 (0.61–0.90)
Similar life-expectancy	7,914 (44.8)	322 (43.2)	4.1	Ref.
Shorter life-expectancy	1,690 (9.6)	108 (14.5)	6.4	1.62 (1.28–2.05)
Do not know	2,753 (15.6)	139 (18.6)	5.0	1.13 (0.91–1.39)
Social support accompanying medical appointments				
Good support	16,069 (91.0)	675 (90.5)	4.2	Ref.
Poor support	1,590 (9.0)	71 (9.5)	4.5	0.99 (0.76–1.30)
Coping scores*	mean ± SD	mean ± SD		Adj. OR (95% CI)
Approach	22.5 ± 4.1	22.5 ± 4.2		1.03 (1.01–1.05)
Avoidance	15.9 ± 5.1	15.8 ± 5.4		0.99 (0.97–1.00)
Diversion	8.1 ± 3.0	8.2 ± 3.2		0.99 (0.97–1.02)
Resignation	7.8 ± 3.0	7.6 ± 3.1		0.98 (0.95–1.00)

High avoidant coping in the recently diagnosed sample: percentages and estimated odds ratios (OR) and corresponding 95% confidence intervals (CI) based on a multivariable logistic regression model, estimating the associations between participant characteristics and high avoidant coping. Descriptive characteristics are reported as counts, column percentages (*n* (column %)), and row percentages (row %) for categorical variables. Row percentages refers to the proportion of the recently diagnosed sample relative to the total study sample for each characteristic. Continuous variables are presented as means with standard deviations (mean ± SD). Associations between characteristics and high avoidance are presented as odds ratios (OR) with corresponding 95% confidence intervals (95% CI), derived from a multivariable logistic regression model, adjusted (adj.) for age, sex, living situation, ethnicity, educational level, and multimorbidity index. “Ref” denotes the reference category used in the logistic regression model. *No missing for coping scores (mean based on *n* = 746 for the total and *n* = 100 individuals with high avoidance).

### 3.2 Avoidant coping in the recently diagnosed sample

High avoidance was operationalised as a total avoidance score exceeding 21.04. Based on this threshold, 13.4% of those in the recently diagnosed sample had a high avoidance score. Descriptive characteristics of individuals with high avoidance in the recently diagnosed sample is presented in Table 2. Descriptive characteristics of individuals with high avoidance in the total study sample is available in Supplementary Table S4.

**Table 2:** High avoidant coping in the recently diagnosed sample and associations with participant characteristics.

Variables	Recently diagnosed sample		
	Total	Individuals with high avoidance	Association between covariates and high avoidance
	*n*	*n* (row %)	Adj. OR (95% CI)
Total	746	100 (13.4)	
Sex			
Female	292	51 (17.4)	Ref.
Male	453	49 (10.8)	0.60 (0.39–0.94)
Age (years)			
50–59	119	11 (9.2)	Ref.
60–69	237	32 (13.5)	1.40 (0.67–2.93)
70–79	291	41 (14.1)	1.49 (0.73–3.06)
80+	99	16 (16.2)	1.61 (0.68–3.80)
Living situation			
Cohabitating	537	66 (12.3)	Ref.
Living alone	209	34 (16.3)	1.15 (0.71–1.84)
Ethnicity			
Danish	715	95 (13.3)	Ref.
Immigrants/descendants	31	5 (16.1)	1.46 (0.53–4.02)
Educational level			
Low	83	18 (21.7)	Ref.
Medium	399	56 (14.0)	0.67 (0.36–1.25)
High	264	26 (9.8)	0.45 (0.23–0.90)
Multimorbidity index			
No multimorbidity	352	38 (10.8)	Ref.
Low	135	19 (14.1)	1.26 (0.69–2.29)
Medium	98	16 (16.3)	1.45 (0.76–2.76)
High	161	27 (16.8)	1.55 (0.90–2.66)
Self-rated health			
Excellent	156	11 (7.1)	0.52 (0.26–1.04)
Good	341	47 (13.8)	Ref.
Poor	249	42 (16.9)	1.19 (0.74–1.91)
Functional capacity			
Good functional capacity	445	51 (11.5)	Ref.
Fair functional capacity	214	32 (15.0)	1.24 (0.76–2.03)
Poor functional capacity	87	17 (19.5)	1.62 (0.86–3.05)
Concern for own health			
Not/slightly concerned	442	51 (11.5)	Ref.
Moderately concerned	163	24 (14.7)	1.29 (0.75–2.20)
Highly concerned	141	25 (17.7)	1.59 (0.92–2.73)
Life-expectancy			
Longer life-expectancy	177	22 (12.4)	1.18 (0.65–2.13)
Similar life-expectancy	322	38 (11.8)	Ref.
Shorter life-expectancy	108	21 (19.4)	2.09 (1.13–3.88)
Do not know	139	19 (13.7)	1.14 (0.62–2.09)
Social support accompanying medical appointments			
Good support	675	81 (12.0)	Ref.
Poor support	71	19 (26.8)	2.64 (1.43–4.90)
Coping scores*	mean ± SD	mean ± SD	Adj. OR (95% CI)
Approach	22.5 ± 4.2	23.0 ± 4.8	1.04 (0.99–1.10)
Avoidance	15.8 ± 5.4	24.2 ± 2.3	-
Diversion	8.2 ± 3.2	12.4 ± 1.7	-
Resignation	7.6 ± 3.1	11.9 ± 1.8	-

High avoidant coping in the recently diagnosed sample: percentages and estimated odds ratios (OR) and corresponding 95% confidence intervals (CI) based on a multivariable logistic regression model, estimating the associations between participant characteristics and high avoidant coping. Descriptive characteristics are reported as counts and row percentages (*n* (row %)) for categorical variables, and as means with standard deviations (mean ± SD) for continuous variables. Associations between characteristics and high avoidance are presented as odds ratios (OR) with corresponding 95% confidence intervals (95% CI), derived from a multivariable logistic regression model, adjusted (adj.) for age, sex, living situation, ethnicity, educational level, and multimorbidity index. “Ref” denotes the reference category used in the logistic regression model. *No missing for coping scores (mean based on *n* = 746 for the total and *n* = 100 individuals with high avoidance).

Results from the multivariable logistic regression model showed that among individuals with a recent diagnosis, being male (OR 0.60; 95% CI 0.39–0.94) and having a high educational level (OR 0.45; 95% CI 0.23–0.90) was significantly associated with reduced odds of high avoidance. In contrast, self-reported shorter life-expectancy and poor social support accompanying medical appointments was significantly associated with increased odds of high avoidance (OR 2.09; 95% CI 1.13–3.88, and OR 2.64; 95% CI 1.43–4.90, respectively). Approach coping scores were similar between individuals with high avoidance coping and the sample with all recently diagnosed individuals (Table 2).

### 3.3 Prediction of high avoidant coping from health parameters and socioeconomics

The total study sample was used as the development dataset for the model. Different prediction models were estimated (Table 3). Baseline Model A with only age and sex, model B with focus on register-available information, and model C with self-reported information from questionnaires.

**Table 3:** Multivariable logistic regressions of the associations between covariates and avoidant coping.

Variables	Total study sample *n* = 17,659			Recently diagnosed sample *n* = 746		
	Model A OR (95%CI)	Model B OR (95%CI)	Model C OR (95%CI)	Model A OR (95%CI)	Model B OR (95%CI)	Model C OR (95%CI)
Sex						
Female	Ref.	Ref.	Ref.	Ref.	Ref.	Ref.
Male	0.66 (0.61–0.73)	0.67 (0.62–0.74)	0.67 (0.61–0.73)	0.56 (0.36–0.86)	0.60 (0.39–0.94)	0.56 (0.36–0.87)
Age (years)						
50–59	Ref.	Ref.	Ref.	Ref.	Ref.	Ref.
60–69	0.88 (0.79–0.97)	0.83 (0.75–0.93)	0.91 (0.82–1.02)	1.54 (0.74–3.18)	1.40 (0.67–2.93)	1.42 (0.68–2.97)
70–79	0.97 (0.87–1.09)	0.87 (0.78–0.98)	1.06 (0.94–1.19)	1.73 (0.85–3.51)	1.49 (0.73–3.06)	1.63 (0.78–3.40)
80+	1.30 (1.09–1.54)	1.05 (0.88–1.26)	1.42 (1.18–1.70)	1.99 (0.87–4.53)	1.61 (0.68–3.80)	1.82 (0.76–4.37)
Living situation						
Cohabitating		Ref.			Ref.	
Living alone		1.18 (1.07–1.30)			1.15 (0.71–1.84)	
Ethnicity						
Danish		Ref.			Ref.	
Immigrants/descendants		1.58 (1.32–1.90)			1.46 (0.53–4.02)	
Educational level						
Low		Ref.			Ref.	
Medium		0.65 (0.56–0.74)			0.67 (0.36–1.25)	
High		0.37 (0.32–0.43)			0.45 (0.23–0.90)	
Multimorbidity index						
No multi-morbidity		Ref.			Ref.	
Low		1.06 (0.93–1.19)			1.26 (0.69–2.29)	
Medium		1.47 (1.27–1.69)			1.45 (0.76–2.76)	
High		1.55 (1.35–1.76)			1.55 (0.90–2.66)	
Self-rated health						
Excellent			0.70 (0.62–0.79)			0.50 (0.24–1.06)
Good			Ref.			Ref.
Poor			1.02 (0.89–1.18)			0.94 (0.52–1.70)
Functional capacity						
Good functional capacity			Ref.			Ref.
Fair functional capacity			1.29 (1.14–1.45)			1.10 (0.63–1.93)
Poor functional capacity			1.47 (1.23–1.76)			1.27 (0.60–2.70)
Concern for own health						
Not/slightly concerned			Ref.			Ref.
Moderately concerned			1.13 (1.00–1.29)			1.02 (0.56–1.87)
Highly concerned			1.41 (1.20–1.66)			1.24 (0.62–2.47)
Life expectancy						
Longer life-expectancy			0.74 (0.65–0.84)			1.39 (0.74–2.60)
Similar life-expectancy			Ref.			Ref.
Shorter life-expectancy			1.51 (1.31–1.75)			1.56 (0.79–3.11)
Do not know			1.04 (0.91–1.17)			1.03 (0.56–1.92)
Social support accompanying medical appointments						
Good support			Ref.			Ref.
Poor support			1.38 (1.21–1.58)			2.40 (1.32–4.36)

Multivariable logistic regressions of the associations between covariates and avoidant coping for the total study sample and the recently diagnosed sample. Model A: adjusted for age and sex; Model B: adjusted for age, sex, living situation, ethnicity, educational level, and multimorbidity index; Model C: adjusted for age, sex, self-rated health, functional capacity, concern for own health, life-expectancy, and social support during medical appointments. OR: Odds Ratio; CI: Confidence Interval. “Ref” denotes the reference category used in the logistic regression model.

### 3.4 Performance of prediction models

**Figure 2:**
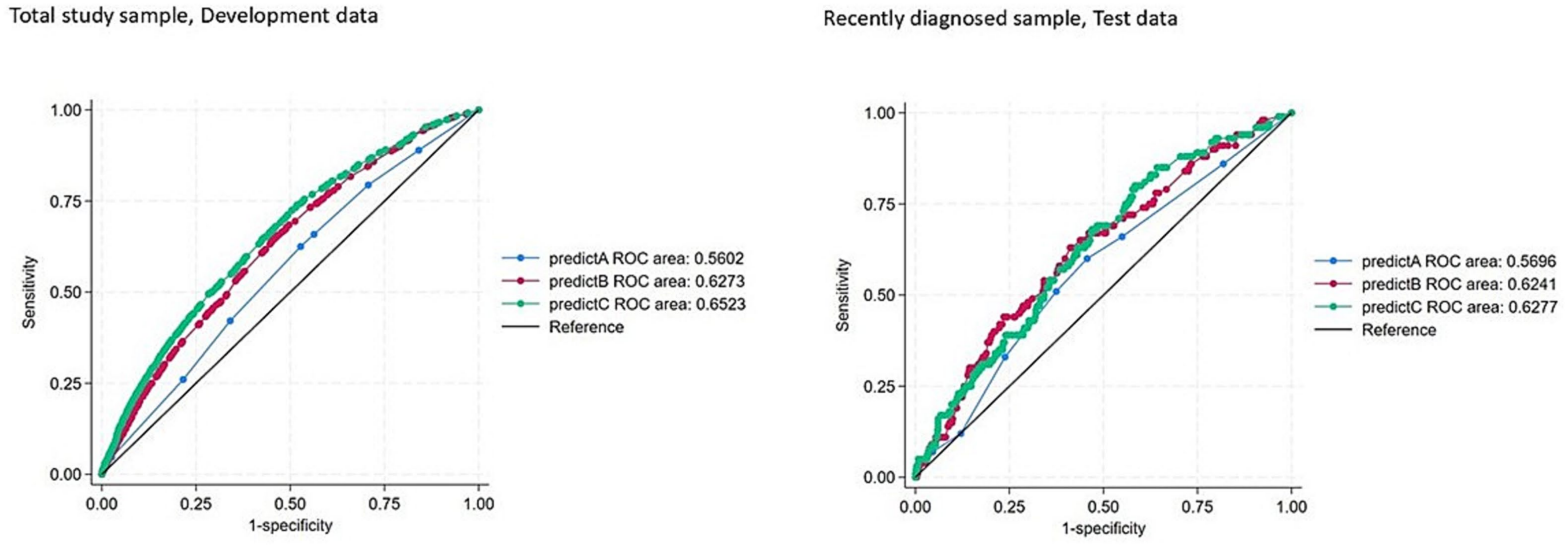
Receiver operating characteristic (ROC) curves for the predicted probabilities. The figure shows Model A (blue line), Model B (red line) and Model C (green line) in the total study sample and the recently diagnosed sample.

The ROC curves for the predicted probabilities for predictive model A, B, and C are available separately for the two populations in Figure 2.

The area under the ROC (95% CI) in the sample with recently diagnosed individuals was 0.57 (0.51–0.63), 0.62 (0.57–0.68), and 0.63 (0.57–0.68) for model A, B, and C, respectively (Figure 2), indicating poor discriminative ability for all three models (Hosmer et al., 2013). Test of model performance is available in Table 4. Adding more covariates to Model A did not improve the predictive value of the model (Model Chi^2^), and the difference in discrimination slopes of the models (IDI) and the scaled Brier Score was low (Table 4). Box plot of predicted probabilities is available in Supplementary Figure S1.

**Table 4:** Performance of the logistic regression models on the total study and the recently diagnosed sample.

Performance measures	Total study sample			Recently diagnosed		
	Model A	Model B	Model C	Model A	Model B	Model C
Model Chi-squared	100.50	413.85	601.93	6.14	15.69	14.61
*p*-value for Likelihood ratio test, A vs. B, A vs. C	1	0	0	1	0.22	0.58
Scaled brier score, %	0.57	2.29	3.54	1.72	2.90	2.65
Discrimination						
AUC (95% CI)	0.56 (0.55–0.57)	0.63 (0.62–0.64)	0.65 (0.64–0.66)	0.57 (0.51–0.63)	0.62 (0.57–0.68)	0.63 (0.57–0.68)
Integrated Discrimination Improvements, IDI, %	0.00	1.81	3.05	0.00	1.71	2.42
Classification						
Sensitivity, %	42.12	46.97	50.06	33.00	51.00	58.00
Specificity, %	65.92	68.88	70.76	76.16	66.10	59.60
Positive Predictive Value, %	16.23	19.13	21.16	17.65	18.89	18.18
Negative Predictive Value, %	87.90	89.23	90.04	88.01	89.71	90.16

Performance of the different logistic regression models on the total study sample (development data) and the recently diagnosed sample (subsample as test data). Model A: Adjusted for age and sex; Model B: adjusted for age, sex, living situation, ethnicity, educational level and multimorbidity index; Model C: adjusted for age, sex, self-rated health, functional capacity, concern for own health, life-expectancy, and social support during medical appointments. AUC: Area Under the Curve; CI: Confidence Interval.

## 4 Discussion

In the present study, we identified a sample of 746 individuals aged 50 years or older who were recently diagnosed with a serious illness. These individuals were more likely to be male, of higher age, with higher levels of multimorbidity and have poorer self-reported health and life-expectancy compared with individuals 50 + years without a recent diagnosis. Based on a threshold defined in the total population, 13% of individuals with a recent diagnosis of serious illness demonstrated high levels of avoidant coping. Odds for high avoidance was lower among males and in individuals with a higher educational level, and higher odds for high avoidance were identified in individuals with shorter life-expectancy and individuals with poor social support accompanying medical appointments. Nevertheless, predictive models based on health parameters and socioeconomics demonstrated poor descriptive ability to predict high avoidant coping.

Avoidant coping was investigated in a population group ranging in age from 50 to over 80 years. The characteristics of individuals being diagnosed with a serious disease were as anticipated based on the literature and clinical experience (Townsend et al., 2022; Deuschl et al., 2020; Dyba et al., 2021). Being recently diagnosed with a serious disease was significantly associated with older age. Additionally, this group was characterised by higher odds of being male, reduced functional capacity, increased health-related concerns, higher level of multimorbidity, poorer self-rated health, and shorter life expectancy.

No significant association was observed between age and high avoidance among individuals recently diagnosed in our study. Gerontopsychological research indicates that older adults typically report experiencing fewer stressors despite facing more pronounced health challenges (Aldwin et al., 2021). This may reflect that older adults experience reduced exposure to stress-inducing roles, such as employment and active parenting, which are not fully offset by health-related stressors (Aldwin et al., 2021). Alternatively, it may be attributed to accumulated life experiences and greater self-awareness, which contributes to effective and less demanding coping strategies, thereby reducing reliance on avoidant behaviours (Aldwin et al., 2021). Hence, based on the literature a negative association between age and avoidant coping among recently diagnosed individuals could be expected. However, as mentioned earlier, this was not supported by our data.

The study demonstrated that poor social support was associated with higher odds for using high avoidance coping. The importance of having strong social networks and support has been highlighted in previous research involving patients with neurological disorders, where individuals who received social support demonstrated higher levels of active coping, planning, and the use of both emotional and instrumental support strategies (Culicetto et al., 2024). These coping behaviours were, in turn, associated with improved quality of life (Culicetto et al., 2024). Interestingly, while social support was significantly associated with avoidant coping, no such relationship was observed between living situation and coping. This distinction underscores the need to differentiate between merely living alone and the presence of supportive individuals during medical encounters. Our findings suggest that the availability of supportive others in clinical contexts may exert a more substantial influence on coping behaviours than general living arrangements. This highlights the importance of considering the quality and context of social interactions rather than relying solely on structural indicators such as household composition when assessing psychosocial determinants of coping.

Shorter perceived life expectancy was associated with higher odds of using avoidant coping. It is important to note that life expectancy was a self-reported item, where respondents were asked to indicate whether they believed they would live longer or shorter than the average person (Supplementary Table S2). For individuals with a serious illness who perceive their life expectancy to be shorter, some degree of avoidance may potentially serve a short-term adaptive function by helping to regulate emotional distress or maintain day-to-day functioning. This nuance warrants further exploration in future research. Our findings revealed notable sex differences in coping behaviour. Males constituted the majority of the recently diagnosed sample, yet they exhibited significantly lower odds of high avoidant coping compared with females. These findings align with previous research conducted in younger non-disease-specific populations (Panayiotou et al., 2017; Matud, 2004) and cancer patients (Oppegaard et al., 2020) where females reported higher levels of avoidant coping.

We developed the prediction models using the total study sample to use a larger sample size and ensure algorithm stability. This approach was based on the hypothesis that the identification of high avoidance would be broadly comparable between the total sample and the subsample of recently diagnosed individuals. We assumed that coping behaviour would not be markedly altered within the applied timeframe following a new diagnosis. Although sex and some health parameters and socioeconomic variables were associated with high avoidant coping, these factors did not nearly capture the complexity of coping responses. The calculated ROC curves all had poor descriptive ability and hence could not reliably predict who will be at risk of using high avoidant coping among individuals with a recent serious illness. Coping is shaped by a complex interplay of factors, including attachment style, personality, bodily sensations, emotional responses, physical symptoms, cognitive processes, available resources, motivation, and overall health status (Leventhal et al., 2016; Aldwin et al., 2021; Panayiotou et al., 2017; Maunder and Hunter, 2015). Potentially, other factors beyond the investigated health parameters and socioeconomic variables may strongly influence coping scores.

Although no coping strategy is inherently good or bad, and individuals often employ multiple strategies simultaneous (Sætre et al., 2023), high avoidance coping has been negatively associated with health outcomes (Penley et al., 2002; Livneh, 2019; Panayiotou et al., 2017; Hasan et al., 2022). In the present study, 13.4% of individuals with a recent diagnosis of serious illness had high avoidance. It is, however, important to note that avoidance is not an uni-dimensional construct, but may be conceptualised as either a passive or disengaged way of relating to stressful events (resignation) or as an active orientation away from the stressor, such as denial, diversion or escape (diversion) (Finset et al., 2002). The different dimensions of avoidance are important, particularly to guide clinical practise, and would be meaningful to address in future research.

### 4.1 Strengths and limitations

This study was strengthened by (i) the utilisation of data from the DaSC II survey (Sætre et al., 2024), (ii) the integration with register-based information on diagnoses, medication, and selected health parameters and socioeconomics (Schmidt et al., 2015; Schmidt et al., 2014; Pottegård et al., 2017), and (iii) the application of a validated questionnaire to assess coping behaviour (Sætre et al., 2023; Finset et al., 2002). The robustness of the DaSC II study was enhanced by the large sample size (distributed to 100,000 randomly selected adults) and the rigorous methodological approach (Sætre et al., 2024). Nevertheless, given that the population of interest comprised individuals in potentially vulnerable circumstances due to recent illness and that the main outcome was avoidant coping, the presence of selection bias is likely. This limits the generalisability of our findings, including the applicability of the cut-off point used to define high avoidance, which may not reflect the threshold in the general population.

The existing literature indicates that the time following a new diagnosis is a period with an increased opportunity to modify adherence to long-term treatment (Chapman et al., 2024). To investigate coping within this “window of opportunity” for early intervention, we included individuals who had recently received a diagnosis of cancer, neurological disorders, and/or cardiovascular conditions. Specific ICD-10 codes were selected based on predefined criteria. However, we acknowledge that individuals who meet similar criteria within other disease categories (e.g., pulmonary conditions and rheumatological diseases) were excluded from the present study sample. Additionally, previous experiences with illness may have influenced coping. It is important to note that although participants were recently diagnosed with a serious illness, this may not have been their first diagnosis. Indeed, half of the study population were registered with multimorbidity, and one in five exhibited a high degree of multimorbidity.

The utilisation of register data provides a valuable opportunity to categorise individuals based on selected variables of interest. However, this approach may be constrained by a lack of nuance, potentially overlooking important contextual factors. For instance, variables such as employment status, commitments to voluntary work, and the presence of children living at home were not captured within the categorisation framework applied in the present study.

The cross-sectional design of the present study restricted us from investigating changes in coping following a recent diagnosis of serious illness. While the stability of coping remains a subject of debate (Greenaway et al., 2015; Folkman et al., 1986; Connor-Smith and Flachsbart, 2007), evidence from seriously ill populations such as breast cancer patients and patients with traumatic brain injury suggests that coping may change (Geyer et al., 2015; Scheenen et al., 2017). In contrast, coping appears more stable among healthy working populations (Nielsen and Knardahl, 2014), indicating that coping may be a dynamic construct influenced by health status. Nonetheless, our findings do not allow us to determine whether coping changed following the diagnosis of serious illness. By employing the BACQ, we assessed general coping tendencies rather than situational coping specific to illness. This approach enabled comparison of coping scores across the entire DaSC II study sample regardless of health status. However, it also limits our ability to determine whether the reported coping behaviour is directly related to the stress associated with a recent diagnosis of serious illness. Future longitudinal studies are needed to clarify whether the various dimensions of coping are state or trait in individuals 50 years and older who have recently been diagnosed with serious illness.

### 4.2 Implications

Coping is often conceptualised as a reflection of a person’s resources with regard to his/her psychological adaptation to illness (Finset et al., 2002). In the period following a new diagnosis of serious illness, individuals appear to be more primed to acquire disease management skills (Chapman et al., 2024), and at the same time individuals are also very attentive to their bodily sensations, e.g., related to disease and treatment, experiences that may contribute to their common sense and influence coping behaviour according to the CSM framework (Leventhal et al., 2016; Diefenbach and Leventhal, 1996). Thus, the time following new diagnoses offers a critical opportunity for healthcare professionals to initiate early interventions that may promote the formation of habits, beliefs, and perceptions about illness that may improve long-term adherence and self-care practices. Our findings regarding the characteristics of individuals with high avoidant coping may inform clinical practice by guiding the provision of tailored support. In particular, they highlight the importance of strengthening social support accompanying medical appointments - a context-specific form of support that cannot be inferred from general living arrangements. Our results indicate that living with others does not necessarily equate to receiving meaningful support in clinical health care settings where the presence and engagement of supportive individuals may play a critical role in shaping coping responses and promoting adaptive health behaviours.

Importantly, these findings underscore that coping cannot be reliably predicted from health parameters and socioeconomics alone. Direct assessment of coping is essential to understand how individuals respond to stressors and to identify those who may be relying on maladaptive coping strategies that could compromise long-term health outcomes. Instruments such as the BACQ offer valuable insight into coping behaviour and may support more nuanced, person-centred approaches to care.

## 5 Conclusion

Among individuals aged 50 years and older, those recently diagnosed with a serious illness were more likely to be male, of higher age, exhibit greater multimorbidity, report poorer self-rated health, express heightened concern about their health, and report shorter life expectancy compared with individuals without a recent diagnosis. Approximately 13% of the recently diagnosed sample demonstrated high avoidance. This subgroup was characterised by lower odds of being male and have a higher educational level, and higher odds of self-reported shorter life-expectancy and poor social support accompanying medical appointments.

Predictive models incorporating health parameters and socioeconomics failed to reliably identify individuals with high avoidant coping. This suggests that additional unmeasured factors may influence coping responses. These findings underscore the importance of directly assessing coping strategies in clinical settings to improve identification of individuals who may require targeted support when adapting to the life changes associated with a newly diagnosed serious illness.

## Data Availability

Raw data were generated at the Research Unit of General Practice, Department of Public Health, University of Southern Denmark. The datasets analysed during the current study are available from the corresponding author (SFB) on reasonable request. Requests to access these datasets should be directed to sbuhl@health.sdu.dk.
