# Supplementary material for: Predicting avoidant coping in individuals recently diagnosed with serious illness: a cross-sectional study
Source: Front Psychol. 2026 Mar 9;17:1773360. doi: 10.3389/fpsyg.2026.1773360 (PMC13006617; doi:10.3389/fpsyg.2026.1773360)

Supplementary Material

Table S1. List of ICD-10 diagnoses used for defining the subgroup of individuals recently diagnosed with serious illness

| **ICD-10** | **Diagnosis** |
| --- | --- |
| ***Recent heart disease*** | |
| DI20 | Angina Pectoris |
| DI21 | Acute myocardial infarction |
| DI240 | Coronary thrombosis not resulting in myocardial infarction |
| DI248 | Other forms of acute ischaemic heart disease |
| DI249 | Acute ischaemic heart disease, unspecified |
| DI26 | Pulmonary embolism |
| DI30 | Acute pericarditis |
| DI31 | Other diseases of pericardium |
| DI32 | Pericarditis in diseases classified elsewhere |
| DI33 | Acute and subacute endocarditis |
| DI34 | Nonrheumatic mitral valve disorders |
| DI35 | Nonrheumatic aortic valve disorders |
| DI36 | Nonrheumatic tricuspid valve disorders |
| DI37 | Pulmonary valve disorders |
| DI38 | Endocarditis, valve unspecified |
| DI39 | Endocarditis and heart valve disorders in diseases classified elsewhere |
| DI40 | Acute myocarditis |
| DI41 | Myocarditis in diseases classified elsewhere |
| DI42 | Cardiomyopathy |
| DI43 | Cardiomyopathy in diseases classified elsewhere |
| DI44 | Atrioventricular and left bundle-branch block |
| DI45 | Other conduction disorders |
| DI46 | Cardiac arrest |
| DI47 | Paroxysmal tachycardia |
| DI48 | Atrial fibrillation and flutter |
| DI49 | Other cardiac arrhythmias |
| DI50 | Heart failure |
| DI51 | Complications and ill-defined descriptions of heart disease |
| DI52 | Other heart disorders in diseases classified elsewhere |
| DI71 | Aortic aneurysm and dissection |
| ***Recent neurological disease*** | |
| DI60 | Subarachnoid haemorrhage |
| DI61 | Intracerebral haemorrhage |
| DI62 | Other nontraumatic intracranial haemorrhage |
| DI63 | Cerebral infarction |
| DI64 | Stroke, not specified as haemorrhage or infarction |
| DI65 | Occlusion and stenosis of precerebral arteries, not resulting in cerebral infarction |
| DI66 | Occlusion and stenosis of cerebral arteries, not resulting in cerebral infarction |
| DI67 | Other cerebrovascular diseases |
| DI68 | Cerebrovascular disorders in diseases classified elsewhere |
| DG20 | Parkinson disease |
| DG35 | Multiple sclerosis |
| DG40 | Epilepsy |
| DG45 | Transient cerebral ischaemic attacks and related syndromes |
| DG122G | Amyotrophic Lateral sclerosis |
| ***Recent cancer*** | |
| DC00-DC99 | Not including DC99: local recidiv and DC44: Other skin cancer |

Table S2**.** Overview of coping questionnaire, variables based on questionnaire data and variables based on register data, including wording of the questions and response categories

|  | | | |
| --- | --- | --- | --- |
| **Coping** | | | |
| **Variable(s)** | | **Question(s)** | **Answer categories** |
| **Approach** | *I say so if I am angry or sad.*  *I like to talk with few chosen people when things get too much for me.*  *I make an active effort to find a solution to my problems.*  *Physical exercise is important to me when having problems or when being in stressful situations.*  *I think something positive could come out of my problems.*  *I firmly believe that my problems will decrease (and my situation improves).* | 5 = agree completely  4 = tend to agree  3 = yes and no  2 = tend to disagree  1 = disagree completely |  |
| **Diversion** | | *I try to forget my problems.*  *I put my problems behind me by concentrating on something else.*  *I bury myself in work to keep my problems at a distance.* |  |
| **Resignation** | *I often find it difficult to do something new when having problems or when being in stressful situations.*  *In difficult situations, I am inclined to feel that I have given up.*  *I withdraw from other people when things get difficult.* |  |  |

| **Questionnaire data** | | | | |
| --- | --- | --- | --- | --- |
| **Variable** | **Question** | **Answer categories** | **Operationalised categories** | **Naming of answers** |
| **Self-rated health** | *In general, would you say your health is* | Excellent  Very Good  Good  Fair  Poor. | Excellent/ very good  Good  Fair/poor | Excellent/ very good  Good  Fair/poor |
| **Functional capacity** | *Do you feel well enough to do what you feel like doing?* | Yes, mostly  Yes, sometimes  No, almost never  I don´t know | Yes, mostly  Yes, sometimes  No, almost never /don’t know | Good functional capacity  Fair functional capacity  Poor functional capacity |
| **Life expectancy** | *Do you think that you will live longer or shorter than the average person?* | Longer than the average person  Like the average person  Shorter than the average person  don´t know | Longer than the average person  Like the average person  Shorter than the average person  don´t know | Longer life-expectancy  Similar life-expectancy  Shorter life-expectancy  Don’t know |
| **Concern for own health** | *To what extent are you concerned about your current health?* | Not at all  Slightly  Moderately  Quite a bit  Extremely | Not at all/ slightly  Moderately  Quite a bit/Extremely | Not/slightly concerned  Moderately concerned  Highly concerned |
| **Social support accompanying medical appointments** | *I have at least one person who can come to medical… (Full length of the question cannot be published to the license agreement of the Health Literacy Questionnaire)* | Disagree completely  Disagree  Agree  Agree completely | Disagree completely/ disagree  Agree/ agree completely | Poor support  Good support |
|  | | |  |  |

|  | **Register Data** |  |
| --- | --- | --- |
| **Variable** | **Register** | **Categories** |
| **Sex** | Danish Civil Registration System | Civil Registration System Number  Ending on:   - equal number: female - unequal number: male |
| **Age** | Danish Civil Registration System | Civil Registration System Number   - counted at the time of invitation based on birthday. - Categorised as followed:   50-59 years  60-69 years  70-79 years  80+ years |
| **Living situation** | Danish Civil Registration System | Family type:  Living alone  Cohabitating |
| **Educational level** | Danish Education Register | Low: < 10 years  Medium: 10-15 years  High: >15 years |
| **Ethnicity** | Danish Civil Registration System | Danish  Immigrants or descendants of immigrants |

Table S3. Univariable logistic regression model, estimating the associations between participant characteristics and being recently diagnosed with serious illness

|  | **Total study sample** | **Recently diagnosed sample** | **Associations between descriptive characteristics and recent diagnosis** |
| --- | --- | --- | --- |
|  |  |  |  |
|  | n | n (row %) | Crude OR (95% CI) |
| **Total** | 17659 | 746 (4.2) |  |
| **Sex** |  |  |  |
| Female | 9550 | 293 (3.1) | Ref. |
| Male | 8109 | 453 (5.6) | 1.87(1.61-2.17) |
| **Age (years)** |  |  |  |
| 50 – 59 | 5930 | 119 (2.0) | Ref. |
| 60 – 69 | 6032 | 237 (3.9) | 2.00(1.60-2.50) |
| 70 – 79 | 4562 | 291 (6.4) | 3.33(2.68-4.13) |
| 80+ | 1135 | 99 (8.7) | 4.67(3.55-6.14) |
| **Living situation** |  |  |  |
| Cohabitating | 12870 | 537 (4.2) | Ref. |
| Living alone | 4789 | 209 (4.4) | 1.05(0.89-1.23) |
| **Ethnicity** |  |  |  |
| Danish | 16785 | 715 (4.3) | Ref. |
| Immigrants/ descendants | 874 | 31 (3.5) | 0.83(0.57-1.19) |
| **Educational level** |  |  |  |
| Low | 1578 | 83 (5.3) | Ref. |
| Medium | 9221 | 399 (4.3) | 0.81(0.64-1.04) |
| High | 6860 | 264 (3.8) | 0.72(0.56-0.93) |
| **Multimorbidity Index** |  |  |  |
| No multimorbidity | 11359 | 352 (3.1) | Ref. |
| Low | 2927 | 135 (4.6) | 1.51(1.23-1.85) |
| Medium | 1569 | 98 (6.2) | 2.08(1.65-2.62) |
| High | 1804 | 161 (8.9) | 3.06(2.52-3.72) |
| **Self-rated health** |  |  |  |
| Excellent | 6113 | 156 (2.6) | 0.60(0.50-0.73) |
| Good | 8209 | 341 (4.2) | Ref. |
| Poor | 3337 | 249 (7.5) | 1.86(1.57-2.20) |
| **Functional capacity** |  |  |  |
| Good functional capacity | 12477 | 445 (3.6) | Ref. |
| Fair functional capacity | 3848 | 214 (5.6) | 1.59(1.35-1.88) |
| Poor functional capacity | 1334 | 87 (6.5) | 1.89(1.49-2.39) |
| **Concern for own health** |  |  |  |
| Not/ slightly concerned | 13294 | 442 (3.3) | Ref. |
| Moderately concerned | 2752 | 163 (5.9) | 1.83(1.52-2.20) |
| Highly concerned | 1613 | 141 (8.7) | 2.79(2.29-3.39) |
| **Life-expectancy** |  |  |  |
| Longer life-expectancy | 5302 | 177 (3.3) | 0.81(0.68-0.98) |
| Similar life-expectancy | 7914 | 322 (4.1) | Ref. |
| Shorter life-expectancy | 1690 | 108 (6.4) | 1.61(1.29-2.01) |
| Don´t know | 2753 | 139 (5.0) | 1.25(1.02-1.54) |
| **Social support accompanying medical appointments** |  |  |  |
| Good support | 16069 | 675 (4.2) | Ref. |
| Poor support | 1590 | 71 (4.5) | 1.07(0.83-1.37) |
|  |  |  |  |
|  | mean ± SD | mean ± SD | Crude OR (95% CI) |
| **Coping scores*** |  |  |  |
| Approach | 22.5±4.1 | 22.5±4.2 | 1.00(0.98-1.02) |
| Avoidance | 15.9±5.1 | 15.8±5.4 | 1.00(0.98-1.01) |
| - Diversion | 8.1±3.0 | 8.2±3.2 | 1.01(0.98-1.03) |
| - Resignation | 7.8±3.0 | 7.6±3.1 | 0.98(0.96-1.01) |

Descriptive characteristics are reported as counts and row percentages (*n* (%)) for categorical variables, and as means with standard deviations (mean ± SD) for continuous variables. Associations between characteristics and recent diagnosis are presented as odds ratios (OR) with corresponding 95% confidence intervals (95% CI), derived from multivariable logistic regression models. *No missing for coping scores (mean based on n=17659 for total sample size and n=746 for recently diagnosed sample).

Table S4. Descriptive characteristics of individuals with high avoidant coping in the total study sample and multivariable logistic regression model, estimating the associations between participant characteristics and having high avoidance

|  | **Total study sample** | | |
| --- | --- | --- | --- |
|  | All | Individuals with high avoidance | Association between covariates and high avoidance |
|  | **n** | **n (row %)** | **Adj. OR (95% CI)** |
| **Total** | 17659 | 2393 (13.6) |  |
| **Sex** |  |  |  |
| Female | 9550 | 1496 (15.7) | Ref. |
| Male | 8109 | 897 (11.1) | 0.67 (0.62-0.74) |
| **Age (years)** |  |  |  |
| 50 – 59 | 5930 | 836 (14.1) | Ref. |
| 60 – 69 | 6032 | 753 (12.5) | 0.83 (0.75-0.93) |
| 70 – 79 | 4562 | 613 (13.4) | 0.87 (0.78-0.98) |
| 80+ | 1135 | 191 (16.8) | 1.05 (0.88-1.26) |
| **Living situation** |  |  |  |
| Cohabitating | 12870 | 1638 (12.7) | Ref. |
| Living alone | 4789 | 755 (15.8) | 1.18 (1.07-1.30) |
| **Ethnicity** |  |  |  |
| Danish | 16785 | 2234 (13.3) | Ref. |
| Immigrants/ descendants | 874 | 159 (18.2) | 1.58 (1.32-1.90) |
| **Educational level** |  |  |  |
| Low | 1578 | 347 (22.0) | Ref. |
| Medium | 9221 | 1397 (15.2) | 0.65 (0.56-0.74) |
| High | 6860 | 649 (9.5) | 0.37 (0.32-0.43) |
| **Multimorbidity Index** |  |  |  |
| No multimorbidity | 11359 | 1363 (12.0) | Ref. |
| Low | 2927 | 397 (13.6) | 1.06 (0.93-1.19) |
| Medium | 1569 | 280 (17.8) | 1.47 (1.27-1.69) |
| High | 1804 | 353 (19.6) | 1.55 (1.35-1.76) |
| **Self-rated health** |  |  |  |
| Excellent | 6113 | 506 (8.3) | 0.59 (0.53-0.67) |
| Good | 8209 | 1151 (14.0) | Ref. |
| Poor | 3337 | 736 (22.1) | 1.56 (1.40-1.74) |
| **Functional capacity** |  |  |  |
| Good functional capacity | 12477 | 1342 (10.8) | Ref. |
| Fair functional capacity | 3848 | 710 (18.5) | 1.70 (1.53-1.88) |
| Poor functional capacity | 1334 | 341 (25.6) | 2.34 (2.02-2.70) |
| **Concern for own health** |  |  |  |
| Not/ slightly concerned | 13294 | 1502 (11.3) | Ref. |
| Moderately concerned | 2752 | 485 (17.6) | 1.60 (1.43-1.79) |
| Highly concerned | 1613 | 406 (25.2) | 2.30 (2.01-2.62) |
| **Life-expectancy** |  |  |  |
| Longer life-expectancy | 5302 | 467 (8.8) | 0.65 (0.58-0.73) |
| Similar life-expectancy | 7914 | 1080 (13.6) | Ref. |
| Shorter life-expectancy | 1690 | 421 (24.9) | 2.08 (1.82-2.38) |
| Don´t know | 2753 | 425 (15.4) | 1.02 (0.90-1.15) |
| **Social support accompanying medical appointments** |  |  |  |
| Good support | 16069 | 2065 (12.9) | Ref. |
| Poor support | 1590 | 328 (20.6) | 1.63 (1.42-1.87) |
|  | **mean ± SD** | **mean ± SD** | **Crude OR (95% CI)** |
| **Coping scores*** |  |  |  |
| Approach | 22.5±4.1 | 22.6±4.4 | 1.02 (1.01-1.03) |
| Avoidance | 15.9±5.1 | 24.0±2.1 | - |
| - Diversion | 8.1±3.0 | 12.1±1.7 | 2.52 (2.43-2.61) |
| - Resignation | 7.8±3.0 | 12.0±1.8 | 2.56 (2.46-2.65) |
| Descriptive characteristics are reported as counts and row percentages (*n* (row %)) for categorical variables, and as means with standard deviations (mean ± SD) for continuous variables. Associations between characteristics and high avoidance are presented as odds ratios (OR) with corresponding 95% confidence intervals (95% CI), derived from multivariable logistic regression models. All models were adjusted (adj.) for age, sex, living situation, ethnicity, educational level, and multimorbidity index. *No missing for coping scores (mean based on n=17659 for total sample size and n=2393 for individuals with high avoidance). | | | |

Figure S1. Box plots of predicted probabilities without (left box of each pair) and with (right box of each pair) high avoidant coping for the Development data (total study sample) and Test data (recently diagnosed sample) in the upper and lower rows, respectively. Model A was adjusted for age and sex; Model B: adjusted for age, sex, living situation, ethnicity, educational level, and multimorbidity index; Model C: adjusted for age, sex, self-rated health, functional capacity, concern for own health, life-expectancy, and social support during medical consultations.


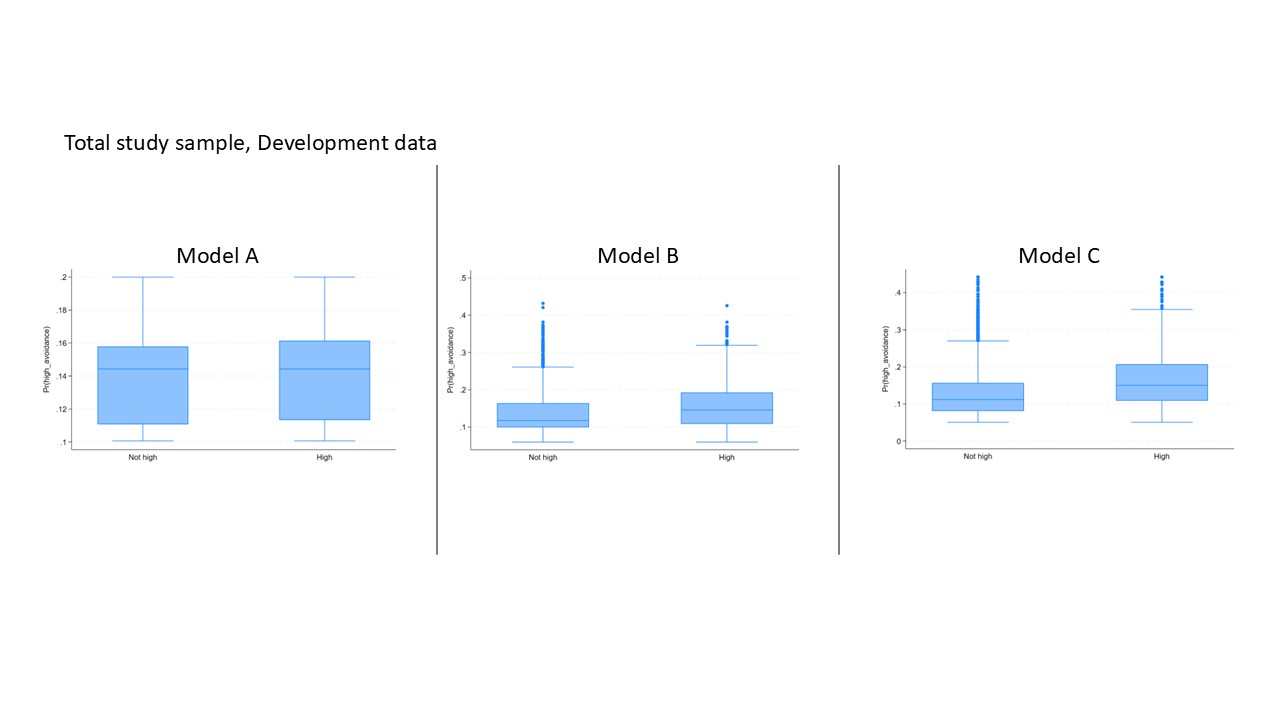

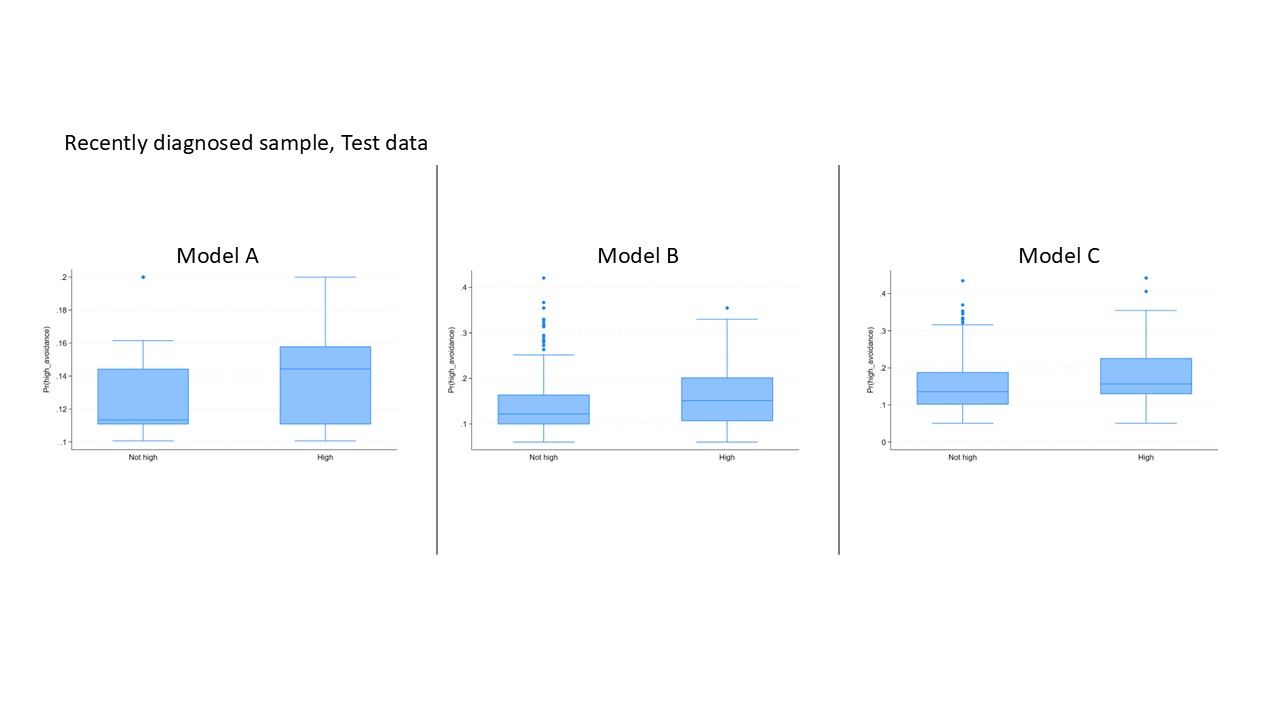

Supplement: Supplementary file 1 [file Table_1.DOCX]
